# Attack Coverage in High-Level Men’s Volleyball: Organization on the Edge of Chaos?

**DOI:** 10.1515/hukin-2015-0080

**Published:** 2015-10-14

**Authors:** Lorenzo Laporta, Pantelis Nikolaidis, Luke Thomas, José Afonso

**Affiliations:** 1 University of Porto, Faculty of Sport (Portugal).; 2 Hellenic Army Academy, Department of Physical and Cultural Education (Athens, Greece).; 3 Top Flight Volley Ltd (England).

**Keywords:** performance analysis, game patterns, systematic approach

## Abstract

Change is pervasive, but emerging patterns are occasionally detectable through analysis of systemic behaviors. Match analysis uses these patterns in order to reduce the degree of improvisation and to optimize the training process. However, it is possible that certain game phases elude systematic patterning. In this vein, our aim was to analyze the case of attack coverage in men’s volleyball, as we suspected it would elude systematic patterning and has received negligible attention in scientific research. We analyzed the occurrence of attack coverage in 4544 plays of the 2011 Volleyball World League. A Chi-square test with residual adjusted values was applied to explore significant associations between variables. A Monte Carlo correction was applied, as some cells had n<5. Effect sizes were determined using Cramer’s V. Overall, attack coverage occurred in 3.89% of ball possessions, and 23 distinct structures emerged. These structures lacked significant associations with the game complex, setting zone, and effect of attack coverage. Conversely, attack coverage structures showed significant associations with the attack zone and tempo, with very strong effect sizes (V=0.472 and V=0.521, respectively). As certain attack zones are deeply associated with attack tempo, it is apparent that quicker attack plays affect attack coverage structuring, promoting the formation of less complex structures. Ultimately, attack coverage structures seem to depend on momentary constraints, thereby rendering rigid systematization impracticable. Still, we contended that a principle-based approach might be suitable. This invites researchers to rethink how to interpret game regularities.

## Introduction

Complex systems have sets of elements in dynamic relation following a common goal (Godinho et al., 1999; Monteiro, 2006; Walter et al., 2007) and maintaining a communication flux and energy exchange (Gréhaigne et al., 1997; Lames and McGarry, 2007; Thelen, 2005; Walter et al., 2007). These interactions result in emerging patterns and their knowledge allows optimizing processes that depend on systemic manipulation, such as training and competition strategies in sports (Lames and McGarry, 2007; McGarry, 2009). Systems can have various subsystems, or even sub-subsystems, which are dependent on the global system, but may be considered separately, as they exhibit a “partial” independency (Gréhaigne et al., 1997; Thelen, 2005). This subsystem autonomy may promote disturbances and instability moments (in most cases non-linear) in the mother system (McGarry, 2009; Walter et al., 2007), generating transitional moments and ultimately leading the system to self-organize in order to return to its previous state or to achieve a new state (McGarry et al., 2002). Whenever the emergent states of a system bring about predictable patterns and structures (Kelso, 1995), it becomes possible to consider manipulating the system to our advantage (Walter et al., 2007). Researchers seek these predictable emerging structures to reduce the role of randomness and improvisation (Bergeles and Nikolaidou, 2011; Lames and McGarry, 2007; Miskin et al., 2010; Peña et al., 2013). However, some sports may present game phases with such highly diversified action possibilities that regular game structures may not emerge. In such cases, prediction of regularities becomes a more risky venture. Still, we contend it might be possible to derive organizational principles regulating such game phases, even if they pave the way to multiple outcomes, thereby inhibiting the emergence of repeatable patterns.

We set out to analyze this phenomenon in volleyball, a team sport of unpredictable and complex nature (Silva et al., 2013). This sport has seen a growing body of scientific research, aiming mostly at increasing game comprehension and improving training strategies and methods (Lames and McGarry, 2007; McGarry, 2009). Researchers use match analysis to enhance the comprehension of the game’s logic (Ciuffarella et al., 2013; Rabaz et al., 2013). Previous systematization of the logic behind the game of volleyball has determined that the game presents distinct complexes (K’s), which present their own set of features and functionality (Bergeles et al., 2009; Palao et al., 2004), and can therefore be separately structured and analyzed (Arias et al., 2011; Hileno and Buscà, 2012). Two complexes appear recurrently in the literature: a) complex I (KI) or side-out, including serve-reception, setting and attack; and b) complex II (KII) or side-out transition, that consists of the serve, block and defense, setting and counter attack (Costa et al., 2012; Costa et al., 2011; Rodriguez-Ruiz et al., 2011). Nonetheless, a comprehensive analysis of the game should also consider complex III (KIII or counterattack to a previous counterattack), complex IV (KIV or attack coverage), and complex V (KV, including freeball and downball) (Arias et al., 2011; Hileno and Buscà, 2012).

**Figure 1:**
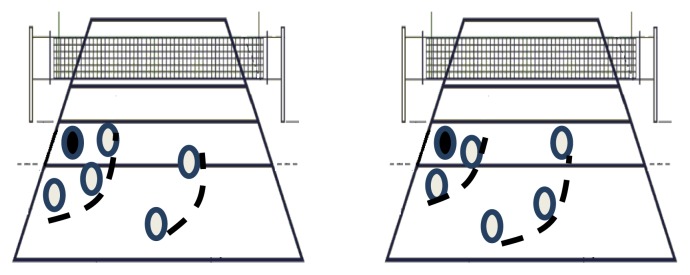
Examples of attack coverage systems described in the literature. Examples of 3:2 and 2:3 attack coverage upon attack in zone 4.

The KIV is characterized by restructuring of the offensive phase after recovering a ball deflected by the block and returning to the attacker’s team (Hileno and Buscà, 2012; Monge, 2003). To the best of our knowledge, only two scientific papers have been published on this topic (Hileno and Buscà, 2012, Laporta et al., 2015), despite being considered a legitimate and relevant game complex. Technical and/or pedagogical books on the topic are outdated, mostly recognizing the existence of only two major attack coverage systems: one with a first defensive line formed by two players and a second line formed by three players (2:3), and another with an inversion of the two lines (3:2) (Asher, 1998; Nicholls, 1973; Selinger and Ackermann-Blount, 1986) (Figure 1). Such simplistic and stereotyped systems seem disengaged from the current complexity of attack models in high-level volleyball, which are increasingly fast and complex (Afonso et al., 2008; Afonso et al., 2005; Castro and Mesquita, 2008; Ciuffarella et al., 2013; Costa et al., 2012), and can exert a major influence on the possible structures during KIV (Hileno and Buscà, 2012).

Therefore, our goal was to verify whether attack coverage affords systematic patterning in high-level men’s volleyball, as well as to analyze the variables influencing those patterns. In the case such patterning is so diversified as to defy systematization, a principle-based approach will be attempted.

## Material and Methods

### Participants

The sample comprised twelve matches of the 2011 Volleyball World League, including the national teams of Argentina, Brazil, Bulgaria, Cuba Italy, Poland, Russia, and the United States of America. A total of 45 sets were analyzed, including 4544 ball possessions, of which 1579 occurred within the context of KI and 2965 within the context of KII.

### Measures

The *game complex* is characterized by the game phase wherein the team is. Complex I (KI or side-out) consists of attack organization after serve-reception, whereas complexes II and III (KII and KIII or counter-attack) include the recovery of the ball and subsequent counter-attack (Castro and Mesquita, 2008; Castro et al., 2011; Mesquita et al., 2010; Silva et al., 2013).

The *setting zone* was evaluated by determining the number of attack options available. We used an adaptation of the model proposed by Esteves and Mesquita (2007), whereas: zone A is localized from the central line until 2 m and away from the right side-line 1 m and 3 m from the left line, allowing all attack options; zone B is localized from the Zone A until 2 m and away the right side-line 1 m and 4 m from the left line, and still affords quick attacks but limits the types of attack combinations; zone C corresponds to the rest of court and affords only setting high balls to the extremities of the court.

The defined *attack zones* were the six official zones of the court (numbered 1 to 6), according to the FIVB rules. As there were a small number of attacks on each of the defensive zones (5, 6 and 1), these three were grouped into a single category termed a *second line*. *Attack tempo* concerns the synchronization between the setter and the attacker; hence, it implies the notion of timing and not merely an absolute time frame. Three attack tempos were adapted from Afonso and Mesquita (2007): 1 (the attacker is in the air before, during, or slightly after the set), 2 (the attacker takes two steps after the set), and 3 (the attacker takes three or more steps after the set).

With respect to the *KIV structure*, we used a field format, i.e., without pre-established categories. It was our purpose to list and describe all the different structures that emerged within the game. For a player to be considered as effectively participating in the attack coverage, he should have his feet on the ground and be facing the attacker and/or blockers (e.g., a setter who jump sets and is still in the air when the attack occurs is not actively involved in attack coverage). The *number of attack coverage lines* consists in the number of lines established by the players, counting from the net to the endline. Finally, *effect of attack coverage* reports to the effect of the play after the occurrence of KIV, namely if it resulted in winning the point, losing the point, of continue playing

### Procedures

The matches were recorded using a high-definition video camera (Sony^®^ Handycam HDR-CX240, 1080p, USA) positioned circa 9 meters in back of the court and at a height of 3 meters, to facilitate video analysis. Data were registered in a worksheet created with Microsoft^®^ Excel^®^ 2013 (Microsoft Office Professional Plus 2013), and later analyzed using IBM^®^ SPSS^®^ Statistics Version 21. The rally’s score, total actions of the teams, the complex in which the team was playing (KI or KII/KIII), setting zone, attack zone and tempo, occurrence of KIV, and KIV structure and effect (i.e., distribution of the players in the court) were registered.

### Statistical analysis

Descriptive statistics were used to show the emerging events and categories. In a first moment, we attempted the construction of predictive models using Multinomial Logistic Regression: *a)* a model predicting the efficacy of attack coverage based on the game complex, setting zone, attack zone, attack tempo, attack coverage structure, and attack coverage lines; *b)* a model predicting the attack coverage structure, based on the game complex, setting zone, attack zone, and attack tempo; and *c)* a model predicting the number of attack coverage lines, also based on the game complex, setting zone, attack zone, and attack tempo. No statistically significant model was achieved, as very strong data dispersion inhibited detection of repetitive game patterns. Immediately after this step, a more modest associative analysis was conducted to scrutinize relationships between the different variables and find meaningful patterns, even if a more global and comprehensive model could not be achieved. Specifically, a Chi-square (**χ**^2^) test with Monte Carlo correction was conducted. If *p* was lower than 0.05, cells containing adjusted residuals above |2.0| were analyzed. Effect sizes were determined by calculating Cramer’s V.

Reliability analysis was conducted for 20% of the occurrences (Tabachnick and Fidell, 2007). Intra-observer reliability was conducted one month after the original observations, and values of Cohen’s Kappa varied between 0.83 and 0.89. An experienced volleyball coach and researcher conducted inter-observer reliability analysis, and values of Cohen’s Kappa ranged from 0.76 to 0.86.

## Results

### Descriptive analysis

Attack coverage occurred in 174 occasions, corresponding to 3.89% of the plays. Of these, 98 occurred after KI (56.3%), and 76 after KII or KIII (43.7%). Twenty-three different KIV structures were observed, grouped into one-line formations, two-line formations, and three-line formations (Table 1).

Coverage systems with one line of coverage represented 2.9% of the total (n=5). Systems with two coverage lines emerged in 51.1% of the cases (n=89), whereas those with three lines corresponded to 46.0% (n=80).

With respect to attack tempo, tempo 1 occurred in 13.2% of the plays preceding attack coverage (n=23), tempo 2 in 62.1% of the plays (n=108), and tempo 3 in 24.7% (n=43). Attack coverage followed attacks in zone 2 in 26.4% of occasions (n=46), zone 3 in 12.1% (n=21), zone 4 in 52.3% (n=91), and second line in 9.2% (n=16).

Concerning the setting zone, 40.8% of attack coverage occurred after setting in zone A (n=71), 31.6% after setting in zone B (n=55), and 27.6% after setting in zone C (n=48). Finally, 19.0% of attack coverage (n=33) and respective counter-attack resulted in the attack error (i.e., they ended up being ineffective), 52.9% allowed to keep the ball in play and prepare another counter-attack (n=92), whereas 28.2% ended up by scoring a point (n=49).

### Associative analysis

When analyzing attack coverage formations, there was a significant association with attack tempo (**χ**^2^=94.418, p≤0.001, V=0.521). Several attack coverage formations associated positively with tempo 1: 1 (3.6), 1//2 (2.2), 1//3 (2.7), 1//4 (2.6), 2//1 (2.8), 3 (2.6), and 4 (2.6). Two formations associated positively with tempo 2: 1//2//1 (2.1) and 2//1//1 (2.1). Finally, one formation associated positively with tempo 3: 1//3//1 (2.4).

There was also a significant association between the attack coverage formation and the attack zone (**χ**^2^=116.197, p≤0.001, V=0.472). Six formations associated positively with attacks in zone 3: 1 (3.8), 1//2 (2.4), 1//4 (2.7), 2//1 (2.9), 3 (2.7), and 4 (2.7). Two formations associated with second line attacks: 3//1 (2.0) and 4//1 (2.9). No significant association was found with the game complex (p=0.263, V=0.385), the setting zone (p=0.433, V=0.359), and effect (p=0.766, V=0.326).

Respecting the number of attack coverage lines, there was an association between the number of lines and attack tempo (**χ**^2^=30.288, p≤0.001, V=0.295). One-line coverage systems associated positively with tempo 1 (4.5) and negatively with tempo 2 (−2.0). Two-line systems associated positively with tempo 1 (2.3). Finally, three-line systems associated negatively with tempo 1 (3.9).

A significant relationship was also found with the attack zone (**χ**^2^=35.152, p≤0.001, V=0.318). One-line systems associated positively with attacks in zone 3 (4.7) and negatively with attacks in zone 4 (−2.4). Two-line systems associated positively with attacks in zone 3 (2.4), whereas three-line systems associated negatively with them (−4.0). Again, there was an absence of association with the game complex (p=0.411, V=0.101), the setting zone (p=0.187, V=0.133), and effect of attack coverage (p=0.749, V=0.074).

## Discussion

Researchers seek to detect predictable game patterns with the aim of improving an understanding of the game and contributing to a more systematized coaching practice (Dutt-Mazumder et al., 2011; Rabaz et al., 2013). The theory of dynamic systems tries to explain the interactions occurring between subsystems and how their behavior can influence the overall system (McGarry et al., 2002; Monteiro, 2006). In this vein, game complexes within volleyball can be considered subsystems, having their own features and internal logic (Arias et al., 2011; Costa et al., 2012). Nonetheless, we argue that certain game phases may present such a highly diversified set of occurrences that prediction of emerging patterns may be rendered impracticable. However, we propose that, in such cases, a principle-based approach might still be useful. Thus, our aim was to verify the possibility of a systematic structuration of KIV, which is poorly researched in the literature.

Results showed the occurrence of under 4% of attack coverage plays, apparently in contradiction with the idea that KIV is relevant to the final outcome as described in technical literature (e.g., Selinger and Ackermann-Blount, 1992). It certainly falls short in comparison with the predominance of actions under complexes I and II/III (Laios and Kountouris, 2005; Marcelino et al., 2010; Zetou et al., 2007; Zetou et al., 2006). Furthermore, circa 19% of attack coverage actions were not effective, while around 28% resulted in a point. Also of notice is the occurrence of 23 distinct KIV structures in only 174 occurrences, denoting the high variability of structures emerging within this game complex, possibly pointing towards a heavy reliance on moment-to-moment constraints. Notwithstanding, some structures were more common than others, namely 2//2 (12.1%), 1//2//2 (10.3%), 2//1//2 (9.2%), and 2//2//1 (12.6%). This is in striking contrast with the simplicity and predominance of the structures highlighted by the literature (e.g. the 2//3 and the 3//2) (Asher, 1998; Selinger and Ackermann-Blount, 1986). In the current sample, the 2//3 represented only 18.4% of the attack coverage occurrences, whereas the 3//2 represented 8%. Thus, a rigid, highly structured approach does not reflect the current characteristics of the volleyball game, at least where attack coverage is concerned. Instead, an approach based on action principles may be more suitable. For example, athletes near the attack action and not involved in other actions should try to cover close to the attacker. Conversely, athletes further away from the attacker should cover the balls that can be directed towards the backcourt, thereby remaining far from the attacker.

Although some game phases of a sport can be highly structured (Gréhaigne et al., 1997; Lames and McGarry, 2007; McGarry et al., 2002), there exist other diversified manifestations in which patterning becomes impracticable. However, our data suggest that some guidelines based on principles are still possible. For purposes of illustration, the association between the attack tempo and structure of attack coverage revealed an effect size of V=0.424, which is considered *worrisomely strong*, meaning both an excellent relationship or that the two variables are measuring almost the same concept. Specifically, tempo 1 is associated with a two-line structure (1//2, 1//4 and 3//1) and with a one-line structure (5). Three or four-line structures were not associated with tempo 1. Thus, although it is difficult to bring about too many conclusions from these scattered data, the fact remains that attack tempo seems to impose a significant restriction on the type of structural organizations that are possible in each sequence of attack coverage. Literature has shown that faster attack tempos associate with less cohesive block opposition and defense (Ciuffarella et al., 2013; Costa et al., 2012). Our data further expand this knowledge, showing that it also undermines the chances of the attacking team to structure a solid attack coverage. High-level volleyball teams have been using quicker and more complex attack plays with the purpose of unbalancing the opponents’ blockers (Ciuffarella et al., 2013; Costa et al., 2012), but this implies a cost in the capacity to cover their own attacks.

As tempo 1 is most commonly used in zone 3 (Afonso et al., 2005; César and Mesquita, 2006; Palao et al., 2007), it comes as no surprise that zone 3 also promoted one-lined coverage systems, again with a worrisomely strong effect size (V=0.472). When only the number of attack coverage lines was considered, the same trends were observed, although with much more modest effect size values (V=0.295 for attack tempo and 0.318 for attack zone). The attack coverage structure was independent of the game complex (I or II/III) and setting zone (A, B, or C). Also, the effect of the attack coverage was independent of the coverage structure. Overall, our results are consistent with those found for attack coverage in high-level women’s volleyball (Laporta et al., 2015).

## Conclusion

Overall, due to its reduced occurrence and lack of effectiveness, attack coverage does not seem to be relevant for achieving a differential in high-level men’s volleyball, except perhaps in highly-balanced matches where few details can establish a difference in the score (Hileno and Buscà, 2012). Furthermore, our data revealed that attack coverage systems traditionally depicted in technical and/or pedagogical books were misleading and did not even approach the complexity and diversity of the game. The wide variation in attack coverage structures denotes its plasticity and dependence upon immediate, momentary constraints. Reaction and intention to cover the attack seem therefore to be more relevant than the specific structure that is used. The association between attack coverage structures and other games variables was scarce, further suggesting a principle-based approach instead of more systematized patterning.

## Figures and Tables

**Figure 2:**
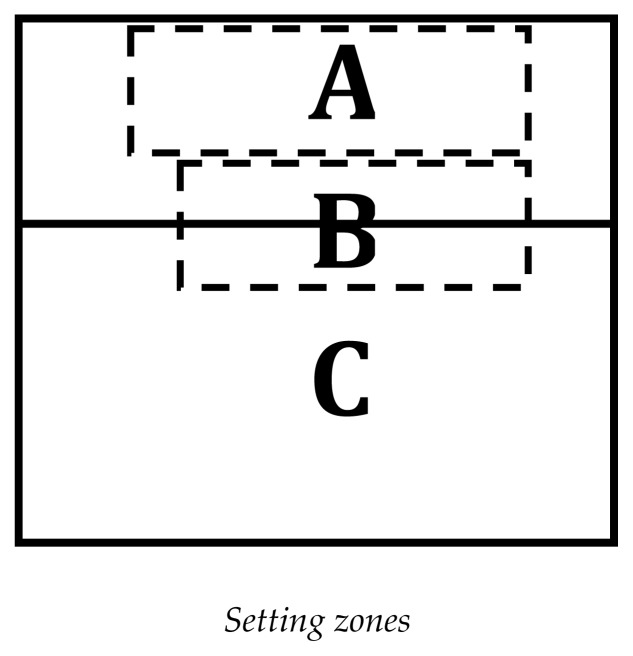
Setting zones

**Table 1:** Attack coverage formations

No. lines	1	2	3
No. structures	4 (1, 2, 3, 4)	10 (1//1, 1//2, 1//3, 1//4, 2//1, 2//2, 2//3, 3//1, 3//2, 4//1)	9 (1//1//1, 1//1//2, 1//1//3, 1//2//1, 1//2//2, 1//3//1, 2//1//1, 2//1//2, 2//2//1)
Frequency of occurrence	5	89	93
Percentage	2.9	51	33.5

Percentage and frequency of attack coverage structures
